# Targeting opioid receptor signaling in depression: do we need selective κ opioid receptor antagonists?

**DOI:** 10.1042/NS20170145

**Published:** 2018-05-14

**Authors:** Sarah J. Bailey, Stephen M. Husbands

**Affiliations:** Drug and Target Discovery, Department of Pharmacy and Pharmacology, University of Bath, Claverton Down, Bath BA2 7AY, U.K.

**Keywords:** antidepressants, depression, opioid receptors

## Abstract

The opioid receptors are a family of G-protein coupled receptors (GPCRs) with close structural homology. The opioid receptors are activated by a variety of endogenous opioid neuropeptides, principally β-endorphin, dynorphins, leu- and met-enkephalins. The clinical potential of targeting opioid receptors has largely focused on the development of analgesics. However, more recent attention has turned to the role of central opioid receptors in the regulation of stress responses, anhedonia and mood. Activation of the κ opioid receptor (KOP) subtype has been shown in both human and rodent studies to produce dysphoric and pro-depressive like effects. This has led to the idea that selective KOP antagonists might have therapeutic potential as antidepressants. Here we review data showing that mixed μ opioid (MOP) and KOP antagonists have antidepressant-like effects in rodent behavioural paradigms and highlight comparable studies in treatment-resistant depressed patients. We propose that developing multifunctional ligands which target multiple opioid receptors open up the potential for fine-tuning hedonic responses mediated by opioids. This alternative approach towards targeting multiple opioid receptors may lead to more effective treatments for depression.

## Introduction

The opioid neuropeptides are β-endorphin, enkephalins and dynorphins, which preferentially act at μ (MOP), δ (DOP) and κ (KOP) opioid receptors respectively [1]. Together with the homologous but non-opioid nociceptin/orphanin FQ (NOP) receptor, these opioid receptors form a subfamily of G-protein coupled receptors (GPCRs) that are expressed throughout the brain and increasingly recognized to play a role in mood and stress responsivity [2,3]. We and others, have focused on developing KOP receptor antagonists with potential as antidepressant and anxiolytic agents [4]. KOP receptors are G_αi/o_ coupled receptors with a unique pharmacology [5]. In this article, we highlight findings that suggest compounds which have activity at a range of opioid receptors may represent a more effective antidepressant strategy by rebalancing hedonic tone.

## Opioid receptors regulate mood

Anhedonia or the reduced capacity to experience pleasure, is a core feature of major depressive disorder [6,7]. The mesolimbic dopaminergic system is involved in the reward system and in mediating the degree of anhedonia and risk of developing depression [6–8]. Hedonic tone can be defined as the trait or genetic predisposition underlying an individual’s baseline range and lifelong characteristic ability to feel pleasure [7]. The activity of the limbic-cortical-striatal-pallidal-thalamic pathway (which consists of connections between the prefrontal cortex, ventro-medial striatum, ventral pallidum, hippocampal subiculum, mediodorsal and midline thalamic nuclei and amygdala) is correlated with hedonic tone in healthy individuals and altered in major depressive disorder [7]. These circuits integrate higher cognitive function with mood and emotional states via reciprocal connections between cortical regions and areas involved in the regulation of autonomic functions such as the periaqueductal grey and the hypothalamus. Neuronal activity in the limbic-cortical-striatal-pallidal-thalamic pathway is predominantly glutamatergic in nature modulated by GABA. Release of both glutamate and GABA can be modulated by opioids in the ventral tegmental area (VTA), amygdala and hippocampus [9]. Dysfunctional reward processing in depression is well characterized and anhedonia has been shown to be a predictor of treatment-resistant depression, particularly in adolescent depression [10,11]. The relative ineffectiveness of SSRIs used to treat anxiety and depression in patients with low hedonic tone is associated with dysfunction of these circuits [12,13]. This would suggest a specific beneficial effect of an antidepressant strategy targeting opioid receptors that could regulate hedonic tone in treatment-resistant depression, and perhaps particularly in adolescent depression where effective treatment options are limited [14,15].

The ‘opium cure’ was recommended for depressed patients before the discovery of current antidepressant treatments or electroconvulsive therapy [16]. Of the endogenous neuropeptide opioids that have been identified as contributing to the aetiology of major depressive disorder, β-endorphin has been the most extensively investigated. However, studies of basal β-endorphin levels in depressed patients have been equivocal with several reports suggesting increased β-endorphin levels compared with controls while others suggest no change or decreased β-endorphin levels [17,18]. This variability may reflect differences between the studies including subgroups of depressed patients, comorbidities and medication status [17]. Despite concerns about the risks of abuse and dependency, opiates acting at MOP receptors, including oxycodone, oxymorphone and the partial MOP agonist buprenorphine, have all been shown to be beneficial in patients with treatment-resistant depression [19–22] and buprenorphine may have antisuicidal effects [23]. In ten patients with treatment-resistant unipolar non-psychotic depression, clinical improvements were evident after 1 week of treatment with buprenorphine (0.15–1.8 mg/day), a significant improvement on existing therapies [19]. More recently, in a small cohort of older treatment-resistant depressed adults, clinically significant improvements were evident within 3 weeks of starting buprenorphine (0.4–0.7 mg/day) treatment [20]. In severely suicidal patients receiving ultra-low-dose buprenorphine (0.1 mg once or twice daily) a greater reduction in Beck Suicide Ideation Score was observed after 2 and 4 weeks of treatment, compared with placebo [21]. Buprenorphine has a complex pharmacology (see below) and its therapeutic mechanisms of action are not well understood. In these studies, different patient populations and different dosing regimes were used but in all cases clinically significant improvements were observed. However, high and moderate efficacy MOP receptor agonists, because of their rewarding properties, are all associated with abuse liability and the potential for developing opiate dependency.

Stress is a risk factor for developing a range of psychiatric disorders including depression [24]. Multiple mediators are implicated in how the body responds to stress including corticotrophin releasing factor (CRF), vasopressin, adrenocorticotrophic hormone and glucocorticoids, which mediate the actions of the hypothalamic-pituitary-adrenal (HPA) axis. Opioids have been suggested to have a counter-regulatory role in modulating HPA stress responsivity under stress conditions [25]. β-endorphin and dynorphin exert tonic inhibition and stimulation of HPA activity by acting on MOP and KOP respectively. β-endorphin acting at MOP exerts tonic inhibition of CRF and thus of the HPA axis in rodents, whereas KOP agonists stimulate plasma corticosterone and these stimulatory effects were blocked by KOP antagonists [26]. Alongside this, accumulating evidence specifically implicates KOP receptors as part of the body’s response to stress [27]. Dynorphin and KOP receptors are expressed in limbic brain regions associated with the regulation of mood. Stress releases CRF which then functions to increase dynorphin release and subsequent activation of KOP receptors in specific brain circuits [28]. In this regard, acute or subchronic stress in rodents produced stress-induced immobility that was reduced by treatment with the KOP receptor antagonist norbinaltorphamine (norBNI) and absent from dynorphin and KOP receptor knockout mice [29–32], confirming that endogenous dynorphins are released and activate KOP receptors during exposure to acute or repeated stress [33]. The mesolimbic dopamine system has been implicated in the blockade of the dysphoric actions of dynorphin and in the antidepressant effects of KOP receptor antagonists [34]. KOP receptors are expressed on VTA cell bodies and on the presynaptic terminals of VTA afferents in the nucleus accumbens and their activation decreases dopamine release, thereby producing a dysphoric effect. Interestingly, direct microinfusion of norBNI into the nucleus accumbens produced an antidpressant effect (Newton et al. (2002) [35]). Together with observations showing that KOP receptor agonists produce dysphoric and psychotomimetic responses in humans [36–39] and aversive responses in rodents [28,35,40], these findings have led to the view that KOP antagonists could be potential antidepressant drugs.

## Clinical trials of selective KOP antagonists

A number of high affinity, selective KOP receptor antagonists have been described in the literature including norBNI, 5′-guanidinonaltrindole (GNTI) and the (3R,4R)-dimethyl-4-(3-hydroxyphenyl) piperidine based JDTic [41–43]. These compounds share an unusual pharmacodynamic property in that there is slow onset of antagonist activity (typically peaking at approximately 24 h) and exceedingly long duration of action *in vivo* (up to 3–4 weeks following a single systemic administration) [44–46]. The first study in humans for JDTic to establish its safety, tolerability and pharmacokinetics was terminated because of adverse effects and specifically non-sustained ventricular tachycardia (https://www.clinicaltrials.gov/ct2/show/NCT01431586). Concerns about the feasibility of developing KOP antagonists for the clinic have centred around the long duration of action of high affinity selective compounds, leading to the development of a number of short-acting KOP antagonists [47–52]. Ligand-directed signalling may account for the duration of activity of KOP antagonists which has been demonstrated to correlate with activation of c-Jun N-terminal kinase-1 (JNK1). The long acting KOP antagonists such as norBNI and JDTic activate JNK1 whereas shorter acting KOP antagonists such as CERC-501 (previously LY-2456302) do not [53,54]. Additionally, there are differences in blood–brain barrier permeability and bioavailability with JDTic showing poor brain penetration, and compounds such as CERC-501 showing relatively rapid absorption. Irrespective of the duration of activity, it is clear that the long-lasting blockade of KOP receptors is not necessary to block stress-induced or pro-depressant responses and indeed the short-acting KOP antagonist CERC-501 has been evaluated in phase II clinical trials in a proof of concept study for the treatment of mood and anxiety (https://www.clinicaltrials.gov/ct2/show/NCT02218736).

## Mixed KOP/MOP antagonists are antidepressant in animal models

We investigated the *in vivo* KOP activity of two naltrindole derivatives which had been identified *in vitro* to have high selectivity for the KOP receptor; 5′-(aminomethyl) naltrindole (5′-AMN) (compound 5, [55]) and the closely related amidine N-((Naltrindol-5-yl) methyl) pentanimidamide (5′-MABN) (compound 10b, [56]). Primary amines are known to be readily metabolizable by amine oxidases and we predicted that these naltrindole derivatives would maintain their selectivity for KOP receptors while having a shorter duration of action than standard KOP antagonists such as norBNI. *In vitro* studies showed that both 5′-AMN and 5′-MABN had high affinity for KOP receptors (*K*_i_: 1.36 ± 0.98 and 0.27 ± 0.08 respectively) and were revealed as potent antagonists at both KOP (*p*A_2_: 7.43 and 8.18 respectively) and MOP receptors (*p*A_2_: 7.62 and 7.85 respectively) in the isolated guinea pig ileum [57]. Systemic administration of both 5′-AMN and 5′-MABN in mice blocked KOP agonist-induced (U50,488) antinociception establishing that they were KOP antagonists. However, they were not short acting and had a duration of action similar to or longer than that of norBNI [57]. Despite this significant MOP antagonist activity, both 5′-AMN and 5′-MABN decreased mouse anxiety- and depression-related behaviours in the elevated plus maze and forced swim test respectively [57]. It was perhaps surprising that concurrent MOP receptor antagonism did not negate the antidepressant effects of KOP receptor antagonism, with both compounds being as effective as the standard selective KOP antagonist norBNI.

An alternative approach to developing short-acting ligands KOP antagonists with antidepressant-like potential was to investigate the effects of combination buprenorphine and naltrexone [58]. Buprenorphine/naltrexone (4 mg sublingual: 50 mg oral) has proved safe and effective in treating opioid dependence in an observational study, in part because it improves the dysphoria associated with drug withdrawal [59]. This would suggest that combination buprenorphine/naltrexone may improve mood. Buprenorphine is a partial MOP receptor agonist, a KOP receptor antagonist and at higher concentrations, a DOP antagonist and also possesses NOP receptor partial agonist activity [60,61]. Naltrexone is a relatively non-selective opioid antagonist with a higher affinity for MOP than KOP receptors. Combining buprenorphine with naltrexone (1:1) produced a functional short acting blockade of both KOP and MOP receptors in CD1 mice as evaluated in the warm water tail withdrawal assay (Almatroudi et al. 2015) [58]. The combination dose of buprenorphine/naltrexone (both 1 mg/kg) produced no locomotor effects, was not rewarding nor aversive but did produce antidepressant and anxiolytic like responses in the forced swim and novelty-induced hypophagia tasks [58]. More recently, we have shown that a novel compound, BU10119, derived from buprenorphine, with a pharmacology resembling combination buprenorphine/naltrexone, also shows antidepressant-like responses [62]. BU10119 was also able to block stress-induced analgesia but not stress-induced increases in corticosterone [62]. Others have shown that stress-induced behaviours can be blocked by KOP antagonists, such as norBNI, even though corticosterone levels may or may not have been affected (see discussion in [62]).

In our experiments, we were also able to demonstrate that naltrexone alone also produced antidepressant-like responses in CD1 mice [58]. The mixed KOP/MOP receptor antagonist profile of naltrexone has recently been proposed to account for its ability to reduce the latency to feed in the novelty-induced hypophagia task in C57BL/6J mice [63]. This was somewhat surprising, since the aversive effects of naltrexone have been known for a long time [64]. While some have shown no effect of naltrexone on mood (daily, 200 mg dose) in overweight healthy volunteers [65], others have shown, in opioid-dependent patients, with a high baseline affective burden, depot naltrexone treatment produced a significant improvement in depression scores [66]. Recently Mischoulon et al. [67] demonstrated that low dose naltrexone (1 mg, twice a day), in a small cohort of patients with recurrent major depressive disorder on dopaminergic antidepressant regimens, showed some benefit as an adjunct therapy, compared with placebo. The present study restricted the patients to those on dopaminergic therapies, predominantly bupropion, because their hypothesis was based on observations in Restless Leg Syndrome which suggested that naltrexone might facilitate sensitization of dopamine D2 and D3 receptors.

As discussed earlier, buprenorphine on its own has demonstrated clinical efficacy in treatment-resistant depression and has also been shown to reduce depressive and anxiety-like behaviours in rats and mice [58,68,69]. There remains concerns about the non-therapeutic misuse of buprenorphine, because of its partial MOP receptor agonist activity, which appears to be rising among drug users [70]. Initially it was believed that buprenorphine’s efficacy in treating depressed patients was derived from its partial MOP agonist actions but this has been challenged in recent years with evidence suggesting these behavioural effects of buprenorphine can be attributed to its KOP receptor antagonism [58,69]. Intriguingly, the most recent data indicate that MOP receptors do play a role in buprenorphine’s behavioural response where there is a motivational component [63,71] but perhaps not in the way initially envisaged. In the novelty-induced hypophagia task in C57BL/6J mice buprenorphine, when used at a time point when it was acting as an MOP receptor antagonist, reduced the latency to approach the food in the novel cage but not in MOP receptor knockout mice. That this activity was mediated through blockade of MOP receptors was confirmed by use of a selective MOP receptor antagonist, cyprodime. These authors suggest that antagonism of MOP receptors by buprenorphine could block stress-induced activation of MOP receptors in the VTA which has been shown to reduce dopaminergic transmission in the nucleus accumbens [63]. It would therefore appear that both KOP receptor antagonism and MOP receptor antagonism may be important for buprenorphine’s ability to regulate emotional state. Clinical evidence is supportive of this hypothesis; Alkermes have combined buprenorphine with the MOP antagonist samidorphan in a single sublingual tablet (ALKS5461) and in a 1:1 combination that provides KOP receptor antagonism and antagonism or extremely low stimulation at MOP receptors. ALKS5461 has been shown to have potential in the treatment of major depressive disorder [72,73] confirming the pre-clinical findings with buprenorphine-naltrexone and BU10119 [58,62].

**Figure 1:**
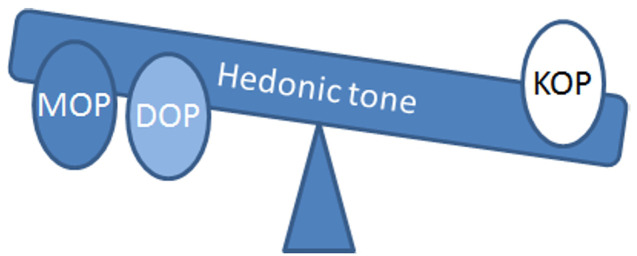
Opioid receptors regulate hedonic tone The opioid receptors MOP, DOP and KOP have all been implicated in the control of hedonic tone. The above diagram illustrates how the balance of activation of these receptors could contribute to overall hedonic tone. Agonist activation of MOP and DOP receptors is proposed to increase hedonic tone whereas activation of KOP receptors produces anhedonia. Blockade of both KOP and MOP receptors by BU10119, buprenorphine, naltrexone, 5′-AMN and 5′-MABN produced antidepressant-like responses in rodents, perhaps by rebalancing hedonic tone.

Overall, these data provide good evidence of the therapeutic potential for exploiting mixed KOP/MOP receptor antagonists as antidepressant treatments, particularly in treatment- resistant depressed patients, to rebalance hedonic tone (Figure 1).

## Future prospects for opioid ligands in the treatment of mood?

In addition to MOP and KOP receptors, DOP and NOP receptors also play a role in the regulation of hedonic tone and the response to stress. Activation of DOP receptors has been shown to be an antidepressant in a number of preclinical behavioural studies [3]. However, DOP agonists may be limited by their pro-seizure-like properties [74,75], although DOP agonists that generate antidepressant-like effects without convulsions have recently been developed [76]. Buprenorphine has no DOP agonist activity, but is a DOP antagonist [61]. The effects of the NOP receptor on stress-related behaviour are less well characterized [77]. NOP receptor agonists have been shown to have anxiolytic-like effects comparable with those of benzodiazepines in a range of behavioural assays [78]. NOP agonists have also been shown to decrease extracellular dopamine in the nucleus accumbens and to block the rewarding and reinforcing properties of morphine and alcohol (Toll et al. (2016) [77]). While buprenorphine and BU10119 show some efficacy at NOP receptors *in vitro*, these effects are evident only at drug concentrations approximately 1000-fold higher than required for MOP and KOP antagonism [61,62,79]. The close structural homologies between the three classic opioid receptors, MOP, DOP and KOP, and the NOP receptor has presented challenges in obtaining conventional selective ligands to study these receptors but will be aided by knowledge of the crystal structure of these GPCRs complexed with ligands [80]. Perhaps bivalent or multifunctional opioid ligands like buprenorphine and BU10119, each with a unique profile of pharmacological activity across the opioid receptors, offer wider therapeutic potential in the treatment of mood disorders [81].

In addition to close structural homology, opioid receptors can function either as a monomer or as part of a homo- or heterodimer or higher multimer [82]. For example, MOP and DOP receptors heterodimerize *in vivo* causing a change in receptor properties and signalling [83]. Both *in vivo* and *in vitro* evidence suggest that these heteromers can switch G-protein coupling preference, thus displaying a significantly different signalling pathway compared with their corresponding homomers [84]. It has also been demonstrated that KOP and DOP receptors heterodimerize *in vivo* in the spinal cord where they produce a unique receptor pharmacology making the design of analgesics with functional selectivity a possibility [85]. Adding further complexity, MOP, KOP and DOP receptors are increasingly recognized to show functional selectivity or biased agonism [86]. This means that ligands which bind to the same receptor can elicit distinct conformations that preferentially signal through distinct G-protein or arrestin subtypes. There is interest in exploiting bias signalling at opioid receptors to improve analgesics as a way of separating the desired therapeutc effect from the unwanted side effects, which limit their clinical utility [87]. The effects of agonist bias signalling through opioid receptors implicated in regulation of mood and anhedonia are largely unknown.

Rebalancing of opioid receptor dysregulation in stress-induced mood disorders is not as simple as targeting a single opioid receptor. There is much potential for designing multifunctional opioid ligands with a unique pharmacology targeting multiple opioid receptors and activating biased intracellular signalling. Better understanding of the downstream signalling pathways of opioid receptors, the role of heterodimers and ligand bias signalling and their role in hedonic tone and motivated behaviours should lead to promising new treatments for depression.

## Abbreviations

CRF: corticotrophin releasing factor
DOP: δ opioid receptor
GABA: gamma-aminobutyric acid
GPCR: G-protein coupled receptor
HPA: hypothalamus-pituitary-adrenal
JDTic: (3R)-7-Hydroxy-N-[(1S)-1-[[(3R,4R)-4-(3-hydroxyphenyl)-3,4-dimethyl-1-piperidinyl]methyl]-2-methylpropyl]-1,2,3,4-tetrahydro-3-isoquinoline-carboxamide
JNK1: c-jun n-terminal kinase-1
KOP: κ opioid receptor
MOP: μ opioid receptor
NOP: nociceptin/orphanin FQ receptor
norBNI: norbinaltorphamine
SSRI: selective serotonin reuptake inhibitor
VTA: ventral tegmental area
5′-AMN: 5′-(aminomethyl) naltrindole
5′-MABN: N-((Naltrindol-5-yl) methyl) pentanimidamide
